# miR-9-5p alleviates the development of abdominal aortic aneurysm by regulating the differentiation of CD4^+^IL-10^+^T cells via targeting the crosstalk between Nrf2 and NF-κB signaling pathways

**DOI:** 10.55730/1300-0152.2754

**Published:** 2025-06-11

**Authors:** Hongfu LIU, Jinyi ZHANG, Lubin LI, Benxiang YU, Chunlei ZHANG, Wenqiang NIU, Yawen CHENG, Hengyang DONG, Yukun ZHANG, Xinlin LUO, Yanlian XIONG, Yueming WANG

**Affiliations:** 1 Department of Anatomy, School of Basic Medicine, Binzhou Medical University, Yantai, Shangdong Province, China; 2 Xu Rongxiang Regenerative Medicine Research Center, Binzhou Medical University, Yantai, P.R. China; 3 Department of Vascular Surgery, Yantai Yuhuangding Hospital, Yantai, Shangdong Province, China

**Keywords:** Abdominal aortic aneurysm, miR-9-5p, Nrf2, oxidative stress, CD4^+^ T cells

## Abstract

**Background:**

Abdominal aortic aneurysm (AAA), a gradual segmental dilatation of the abdominal aorta, is associated with a high mortality rate. The pathophysiological molecular mechanisms underlying AAA remain unclear. In recent years, changes in miRNA levels have been reported to be involved in the development and treatment of AAA. This study aimed to investigate the potential targets and underlying mechanisms of miR-9-5p in attenuating AAA progression by modulating the inflammatory response.

**Materials and methods:**

Biochemical kits were used to measure the levels of inflammatory factors, antioxidant enzyme activity, and serum oxidative stress in normal and AAA model mice. miR-9-5p overexpression was achieved by transfecting miR-9-5p mimics into CD4^+^ T cells and administering an miR-9-5p agomir to the mice. The effect of miR-9-5p overexpression was evaluated by detecting the expression level of miR-9-5p in CD4^+^ T cells through qRT–PCR. The NF-κB/Nrf2 pathway levels were assessed using immunofluorescence, western blotting, and quantitative PCR. miR-9-5p expression was modulated by transfecting either miR-9-5p mimics or inhibitors, and the impact on CD4^+^IL-10^+^ T-cell differentiation was analyzed using flow cytometry.

**Results:**

Compared with that in the control group, miR-9-5p expression in CD4^+^ T cells from the peripheral blood of AAA model mice was decreased by 28%. In vivo, miR-9-5p intervention reduced AAA formation in model mice and markedly decreased serum oxidative stress damage and inflammatory factor levels. Furthermore, miR-9-5p intervention significantly increased miR-9-5p levels in CD4^+^ T cells both in vitro and in vivo, increased the proportion of CD4^+^IL-10^+^ T cells, suppressed NF-κB expression, and upregulated Nrf2 and its downstream antioxidant genes. Conversely, these therapeutic effects were abolished when an miR-9-5p inhibitor was administered.

**Conclusions:**

By controlling the interaction between the Nrf2 and NF-κB signaling pathways, miR-9-5p mediates the differentiation of CD4^+^IL-10^+^ T cells and alleviates the development of AAA.

## 1. Introduction

Abdominal aortic aneurysms (AAAs) are a leading source of morbidity and mortality in many countries (Sakalihasan et al., 2005). The pathophysiology of AAA is a complex process that includes matrix metalloproteinase activation, oxidative stress, inflammatory responses, smooth muscle death, and extracellular matrix (ECM) degeneration (Sakalihasan et al., 2018; Piacentini et al., 2019). Although the mechanism underlying the pathogenesis of AAA has not been fully elucidated, recent research suggests that the emergence of inflammatory factors caused by immune system metabolic dysfunction is a significant trigger for AAA (Yuan et al., 2020).

While the exact pathophysiology of AAA is unknown, inflammation has been shown to be a major factor. A protease released by inflammatory cells during AAA formation causes the ECM to degrade and reduces the tunica medium’s resilience, which encourages the infiltration and aggregation of inflammatory cells within the tunica media (Cameron et al., 2018). The mediators released by CD4^+^ T cells participate in the pathophysiology of aneurysmal lesions in patients with AAA (Cameron et al., 2018). Several investigations have indicated that the mediators released by CD4^+^ T cells participate in the pathophysiology of aneurysmal lesions in AAA patients (Yin et al., 2010; Téo et al., 2018). The pathophysiology of AAA may be influenced by Treg immunoregulatory issues and increased Th17 and Th22 subsets (Yin et al., 2010). Because of their detrimental immunomodulatory effects, IL-10-producing CD4^+^ T (CD4^+^IL-10^+^ T) cells have recently garnered much interest in the treatment of autoimmune disorders (Zhang et al., 2021). However, the role and the underlying regulatory mechanism of CD4^+^IL-10^+^ T cells in the development of AAA remain to be elucidated.

By attaching itself to its target mRNA, a noncoding microRNA (miRNA) can take part in the control of gene expression. Numerous studies have demonstrated that miRNAs are important in the onset of many diseases, and miRNA targeting has emerged as a promising treatment for a number of illnesses (Zhao et al., 2018; Nasci et al., 2019). Zalewski et al. (2020) discovered disruption of the miRNA modulation network in patients with AAA. Zhang et al. (2020) demonstrated that miR-146a targets CARD10 to control inflammation and development in patients with AAA. Li et al. (2020) demonstrated that miR-126a-5p limits the formation of AAAs in mice. Together, these findings suggest that, by controlling gene expression in individuals with AAA lesions, miRNAs may have a therapeutic effect. Although miR-9-5p can participate in the regulation of the immune microenvironment by regulating Th17 cell differentiation (Shirani et al., 2020), its role in the inflammatory response and immune imbalance of AAA tissue is still unclear.

Thus, the goal of the current work was to investigate the functions of miRNAs that preserve immunological homeostasis by controlling the development of CD4^+^IL-10^+^ T cells, and consequently clinically reduce AAA lesions.

## 2. Materials and methods

### 2.1. Reagents

DAPI(#4083) and antibodies against Histon H3 (# 4499 ), Nrf2 (#12721), NF-κB (#8242), β-actin (# 4970 ), HO-1 (#43966), and NQO1 (#62262) were obtained from CST (Massachusetts, USA). The total antioxidant capacity (TAC) and total oxidant status (TOS) kits were obtained from Rel Assay Diagnostic (Gaziantep, Türkiye). Ang II was obtained from Sigma-Aldrich (t#A9525, Sigma-Aldrich, St. Louis, MO, USA). RNA was extracted using an RNeasy Plus Mini kit obtained from Qiagen (#74134, Qiagen Inc, CA, USA).

### 2.2. Mice and AAA models

Male C57BL/6 mice (8–10 weeks) were obtained from Lvye Pharmaceutical Co., Ltd., Shandong, China (SYXK 2018–0028). Mice had free access to water and chow and were housed in a standard environment with a regular 12/12 h light/dark cycle. This study was performed following protocols approved by the Binzhou Medical University Institutional Animal Care and Use Committee (permit number: 2022-02).

To create the Ang II-induced AAA model, male mice aged 8–10 weeks were given either Ang II or saline injections, as previously mentioned (Liu et al., 2016). In brief, mice were put to sleep by injecting a combination of xylazine (5 mg/kg) and ketamine (100 mg/kg) intraperitoneally. The pedal withdrawal reflex vanished, confirming adequate anesthesia. Next, a small incision was made in the dorsum of the neck to implant a microosmotic pump (Alzet, Model 2004, DURECT Corporation, Cupertino, CA, USA) loaded with Ang II or saline into the subcutaneous region. For 28 days, the injection was administered at a rate of 1 μg/kg/min.

To determine the functional role of miR-9-5p in Ang II-induced AAA, 40 mice were randomly divided into four groups (n = 10): the control group, the AAA group, the AAA+ miR-9-5p agomir group, and the AAA+ agomir NC group. The sample size was determined following the 3R principle (Replacement, Reduction, Refinement) to minimize animal use while ensuring statistical validity. Using the aforementioned techniques, AAA modeling was performed in the control and AAA groups using either saline or Ang II interventions. Following AAA modeling, the AAA+ miR-9-5p agomir group and the AAA+ agomir NC group tail vein injections of either miR-9-5p agomir or agomir NC at the same dosage (1 nmol/g/day) for 4 days (on day0, day3, day7, and day11). The miR-9-5p agomir and agomir NC in this investigation were created and synthesized by a commercial business (RiboBio, Guangzhou, Guangdong, China).

Two weeks after treatment with either miR-9-5p agomir or negative control (NC), the mice were anesthetized with pentobarbital sodium (40 mg/kg, i.p.). Blood was collected from the eye socket, and the mice were sacrificed by cervical dislocation. The entire aorta was harvested, some of them were fixed in 4% paraformaldehyde for histological evaluation, and the others were snap-frozen in liquid nitrogen and stored at −80 °C for subsequent protein and mRNA analysis.

### 2.3. Tissue analysis

In accordance with previous research (Zhou et al., 2021), the entire aorta, from the thoracic aorta to the iliac artery bifurcation, was thoroughly dissected using a microscope. Image Pro Plus software was used to photograph the abdominal aorta segment and measure its maximum diameter. AAA is defined as an increase of more than 50% in abdominal aorta diameter over preintervention levels. The aorta at the site of maximal diameter was sliced and embedded in paraffin after it was preserved for 24 h in 4% paraformaldehyde. Hematoxylin and Eosin (H&E) staining was applied to 5-μm-thick paraffin slices. The stained slices were photographed with a microscope (Leica DM4000, Leica Microsystems, Wetzlar, Germany).

### 2.4. Preparation of single peripheral blood mononuclear cells (PBMCs)

Mice-derived peripheral blood mononuclear cells (PBMCs) in different groups were obtained in accordance with the instructions of the mouse PBMC separation solution kit (P6340, Solarbio, Beijing, China). Briefly, whole blood was diluted with an equal volume of whole blood diluent from the kit, and then 3 mL of mononuclear cell separation solution was added. Next, the solution was centrifuged at 800 × *g* at RT for 30 min. After centrifugation, the mononuclear cell layer was carefully aspirated, resuspended in 10 mL of prechilled PBS, centrifuged at 250 × *g* at 4 °C for 10 min, and resuspended in 500 μL of prechilled PBS with 0.04% BSA.

### 2.5. Differentiation of human CD4^+^IL-10^+^ T cells

Human CD4^+^IL-10^+^T cells were induced in vitro using the previously reported procedures (Huang et al., 2018). Human PBMCs were obtained from healthy donor blood samples (Central Blood Bank in Yantai, China) using gradient separation with Ficoll-Hypaque (1.077 g/mL, Solarbio, Beijing, China). PBMCs were cultured with the RPMI-1640 medium (HyClone, Boston, MA, USA) and incubated at 37 °C. Human naive CD4^+^T cells (CD4^+^CD45RA) were obtained using the CD4^+^T Cell Isolation Kit II (Miltenyi, Bergisch Gladbach, Germany). Lipofectamine 2000 (Invitrogen, Carlsbad, CA, USA) was used to transfect CD4^+^T cells with 50 nM miR-9-5p mimics or mimic negative control. Antihuman CD3ɛ and antihuman CD28 mAb (1 μg/mL, Life Technologies, Waltham, MA, USA), recombinant human (rh) IL-2 (200 U/mL, Proteintech, Wuhan, China), IL-10 (100 U/mL, Proteintech), IL-27 (63 U/μL, Proteintech), and IFN-α2b (2.09 U/μL) were also used. After 3 days, cells were stimulated with PMA/ionomycin (1.25 μg/mL, 0.25 mg/mL, MultiSciences, Hangzhou, China) and BFA/monensin (0.75 mg/mL, 0.25 mg/mL, MultiSciences) for 5 h, followed by surface and intracellular staining techniques. The miR-9-5p mimic and mimic negative control used in this investigation were created and synthesized by a commercial business (RiboBio, Guangzhou, Guangdong, China), and their complete sequences are presented in the Supplementary material.

### 2.6. Flow cytometry

The FACSCanto II flow cytometer (BD Biosciences, CA, USA) was used to measure the proportion of CD4^+^IL-10^+^T cells in PBMCs. Briefly, cells were treated with antibodies against surface markers at 4 °C in the dark for 25 min. The cells were fixed, permeabilized, and then stained with fluorescently tagged antibodies at 4 °C in the dark for a further 25 min to perform intracellular labeling. Antimouse CD3-PerCP-Cy5.5 (BD Biosciences), antimouse CD4-APC (BD Biosciences), and antimouse IL-10-FITC (BD Biosciences) were utilized.

### 2.7. Detection of serum total oxidative stress (TOS)

Erel (2005) devised a method to quantify serum TOS. In short, 35 μL of serum was combined with 225 μL of Reagent 1 (xylenol orange 150 μM, NaCl 140 mM, and glycerol 1.35M in 25 mM H_2_SO_4_ solution, pH 1.75), and each sample’s absorbance was measured spectrophotometrically at 560 nm as a sample blank. The mixture was then mixed for approximately 3–4 min with 11 μL of Reagent 2 (ferrous ion 5 mM and o-dianisidine 10 mM in 25 mM H_2_SO_4_ solution), and the final absorbance was measured at 560 nm. Based on the variations in absorbance at 560 nm before and after the addition of Reagent 2, TOS was computed. The assay was calibrated using H_2_O_2_, and the results were reported in μmol H_2_O_2_ Equiv/L, or μM H_2_O_2_ equivalent per liter.

### 2.8. Detection of serum total antioxidant capacity (TAC)

The serum TAC assay was based on the measurements of the reduction of 2,2′-azino-bis (3-ethylbenzothiazoline-6-sulphonic acid; ABTS) radical (Zhang et al., 2015). Salivary TAC was evaluated by mixing 5 μL of saliva with 225 μL of Assay Reagent 1 (acetate buffer, pH 5.8), and then measuring the absorbance at 420 nm after 30 s of incubation. Following a 5-min incubation period, 20 μL of Reagent 2 (ABTS, 30 mM in acetate buffer, pH 3.6) was added to each sample, and the absorbance at 420 nm was recorded. Based on the variations in absorbance at 420 nm before and after the addition of Reagent 2, TAC was computed. The results were reported in mM trolox equivalent per liter (mmol trolox Equiv/L), after the assay was calibrated using trolox.

### 2.9. Detection of serum cytokines

Mouse sacrifice was followed promptly by the collection of blood from the hearts. In accordance with the manufacturer’s instructions, the Bio-Plex-200 system (Bio-Rad Laboratories, USA) was used with the Pro Mouse Cytokine Panel kit (targets include: IL-6, IL-17A, TNF-α, and IFN-γ) (#M6000007NY, Bio-Rad Laboratories, USA) to analyze the relevant cytokines, including IL-6, IL-10, TNF-α, and IFN-γ, in mouse serum.

### 2.10. Immunofluorescence

To conduct the Nrf2 and NF-κB nuclear translocation assays, cells were fixed with 4% paraformaldehyde, washed, and blocked for 2 h with 1% bovine serum albumin (Solarbio, Beijing, China), 10% normal goat serum, and 0.3% Triton X-100 (Solarbio). Primary antibodies were added, and the cells were treated at 4 °C overnight. Goat antimouse IgG (DyLight 549) and goat antirabbit IgG fluorescently labeled secondary antibodies (DyLight 488) were then added, and samples were incubated at room temperature for 2 h. Finally, a nuclear counterstain, 4,6-diamidino-2-phenylindole (DAPI) (Cell Signaling Technology, MA, USA), was applied to the samples and incubated for 2 min at room temperature. After the staining procedures were completed, photos were taken with a fluorescence microscope (LSM880 with Airyscan, Zeiss, Oberkochen, Germany), and the mean fluorescence intensity values were calculated using the ZEN software. The fluorochromes employed were DAPI, red fluorescent protein, and green fluorescent protein.

### 2.11. Western blotting

We investigated the protein levels of Nrf2, HO-1, NQO1, and NF-κB in CD4^+^ T cells using western blotting. The tissue and cellular protein extracts were separated by SDS-PAGE, and the electrophoresed proteins were transferred to PVDF membranes. Membranes were treated overnight at 4 °C with antibodies to Nrf2, HO-1, NQO1, and NF-κB. The membranes were then washed four times with goat antirabbit and goat antimouse secondary antibodies (Proteintech, Wuhan, China), and the samples were incubated for 2 h at room temperature. The protein bands were observed using a western blot imager after the blots had been washed and produced with an ECL substrate (Beyotime, Nanjing, China).

### 2.12. Dual-luciferase reporter gene assay

TargetScan^1^ predicted a putative binding site between NF-κB and miR-9-5p. A commercial company (RiboBio, Guangzhou, Guangdong, China) manufactured the miR-9-5p mimics/inhibitors and corresponding negative control (NC). Amplicons were introduced into the SacI and XbaI cleavage sites of the pmirGLO vector (E1330, Promega, USA). Human embryonic kidney 293T cells (purchased from Procell Life Science & Technology Co., Ltd., Wuhan, China) were chosen because of their low endogenous miRNA expression. Lipofectamine 2000 (Invitrogen, USA) was used to cotransfect HEK293T cells with 800 ng of wild-type or mutant reporter and 20 μM miR-9-5p mimic/inhibitor. Renilla and Firefly luciferase activity in lysed HEK293T cells was measured 24 h after transfection using the Dual-Luciferase Reporter Assay System (E1910, Promega, USA).

### 2.13. RNA extraction and quantitative real-time PCR

mRNA was extracted using either the TRIzol Reagent (CW0580, CWBio, Jiangsu, China) or the miRNA Purification Kit (CW0560, CWBio, Jiangsu, China). Quantitative real-time PCR was used to quantify miRNA and mRNA levels. Gene relative abundance was estimated using the 2-ΔΔCT formula. U6 was the internal reference for miR-9-5p, while β-actin was the internal reference for mRNA. Primers were included in the Supplementary material.

### 2.14. Statistical analysis

The data was reported as mean ± SEM. Statistical significance was established using an unpaired two-tailed Student’s t-test (or nonparametric test), one-way ANOVA, and Tukey’s test for multiple comparisons (Xiong et al., 2016). A value of p < 0.05 indicated statistical significance.

## 3. Results

### 3.1. miR-9-5p protects against Ang II-induced AAA formation

**Figure 1:**
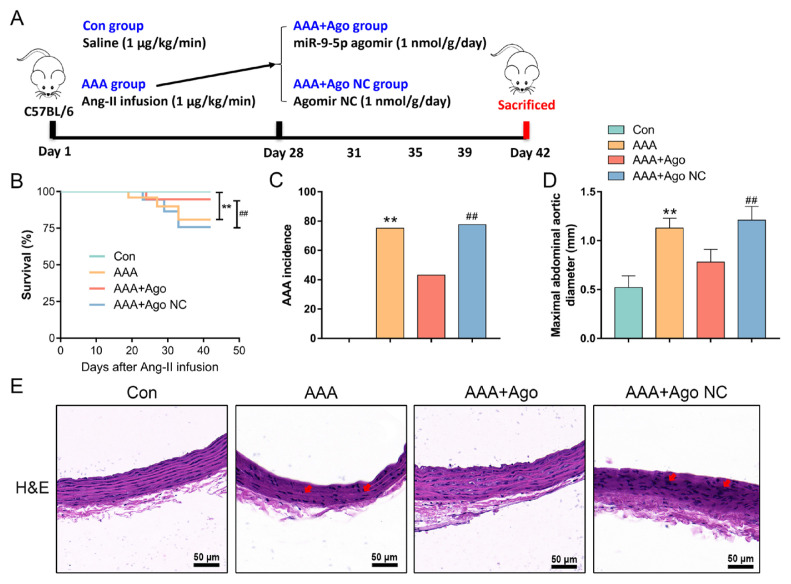
*miR-9-5p protects against Ang II-induced AAA formation*. (A) The scheme of the experimental design. (B) Survival rates after Ang II infusion-induced AAA. (C) Incidence of AAA formation. (D) Maximal abdominal aortic diameter. (E) Representative photomicrographs of H&E-stained renal sections (bar = 50 μm). Data represent the mean scores ± SEM of at least three independent experiments, ^**^p < 0.01, Con (Control) group vs. AAA group; ^##^p < 0.01, AAA+Ago group vs. AAA+Ago NC group.

Following the experimental design outlined in Figure 1A, we conducted animal experiments to investigate the effects of miR-9-5p on Ang II infusion-induced AAA formation and development in mice. As shown in Figures 1B–1D, the AAA group presented a significantly greater incidence of AAA and a greater maximal abdominal aortic diameter than the control group. Concurrently, the survival rate of AAA model mice decreased significantly. In contrast, compared with the agomir NC group, miR-9-5p agomir intervention reduced the incidence of AAA and abdominal aortic dilation while increasing the survival rate (Figures 1B–1D). Additionally, H&E staining revealed that arterial smooth muscle cells in the AAA group were disorganized and infiltrated with inflammatory cells compared with those in the control group (Figure 1E). Notably, this pathological damage to the abdominal aortas of AAA model mice was significantly reversed by miR-9-5p agomir treatment (Figure 1E). These findings suggest that miR-9-5p intervention inhibits the development of AAA induced by Ang II infusion in mice.

### 3.2. miR-9-5p reduces serum oxidative stress injury and the expression of inflammatory factors in AAA lesion model mice

**Figure 2:**
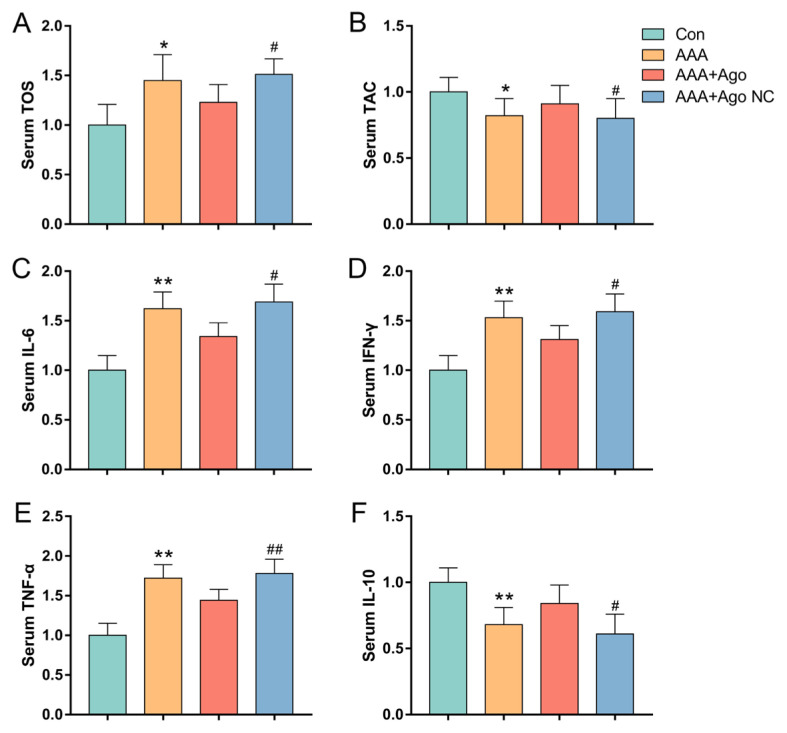
*miR-9-5p attenuates disorder of redox metabolism and inflammatory response in AAA lesion mice*. Levels of serum TOS (A) and TAC (B). Levels of serum cytokines IL-6 (C), IFN-γ (D), TNF-α (E), and IL-10 (F). Data represent the mean scores ± SEM of at least three independent experiments, ^*^p < 0.05, ^**^p < 0.01, Con group vs. AAA group; ^##^p < 0.01, ^#^p < 0.05, AAA+Ago group vs. AAA+Ago NC group. TAC, total antioxidant capacity; TOS, total oxidative stress; IL-6, interleukin-6; IFN-γ, interferon-gamma; IL-10, Interleukin-10; TNF-α, tumor necrosis factor alpha.

Oxidative stress plays a pivotal role in the pathogenesis and progression of AAA. We examined the variations in serum TOS and TAC levels in peripheral blood to assess the regulatory impact of miR-9-5p on the equilibrium between oxidation and antioxidation in AAA lesion model mice. In AAA lesion model mice, the level of serum TOS was significantly higher and the level of serum TAC was significantly lower than those in the control group, as illustrated in Figures 2A and 2B. In contrast, compared with the agomir NC group, miR-9-5p agomir intervention significantly decreased the level of serum TOS and increased the level of serum TAC.

Oxidative stress activates proinflammatory pathways (e.g., the NF-κB and MAPK pathways), promoting the recruitment of immune cells (macrophage and T cells) to the aortic wall. These cells further produce ROS and proinflammatory cytokines (e.g., IL-6 and TNF-α), creating a vicious cycle of inflammation and tissue damage. To further explore the antiinflammatory effects of miR-9-5p, we analyzed the serum cytokine profiles. Compared with the control group, the AAA lesion model mice presented significantly higher levels of proinflammatory cytokines (IL-6, TNF-α, and IFN-γ) and lower levels of antiinflammatory cytokines (IL-10), as illustrated in Figures 2C–2F. Furthermore, compared with the agomir NC group, the miR-9-5p agomir intervention group presented significantly lower levels of IL-6, TNF-α, and IFN-γ and higher levels of IL-10 in AAA lesion model animals. The onset of AAA is accompanied by a significant increase in inflammation. These results suggest that miR-9-5p treatment reduces the inflammatory response and redox metabolism dysfunction in the peripheral blood of mice with AAA lesions.

### 3.3. miR-9-5p regulates the differentiation of CD4^+^IL-10^+^ T cells in AAA model mice

**Figure 3:**
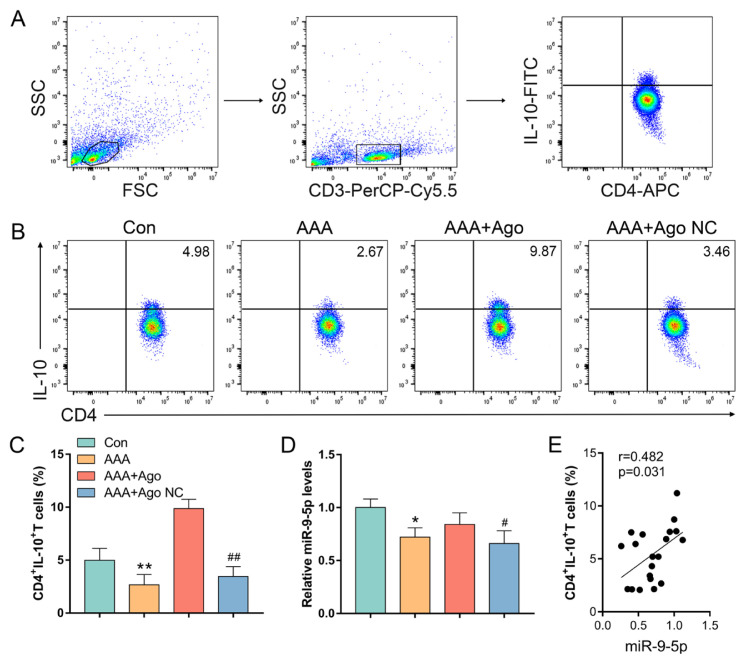
*miR-9-5p regulates the differentiation of CD4^+^IL-10^+^ T cells in AAA lesion mice*. (A) Gating strategy: forward scatter (FSC) and side scatter (SSC) gating were used to discriminate viable cells from cell debris. Within the lymphocyte gate, CD3 was used as a T cell maker. (B) Representative dot plots of CD4^+^IL-10^+^ T cells. (C) The percentage of CD4^+^IL-10^+^ T cells. (D) The expression of miR-9-5p. (E) Pearson’s correlation analysis between the levels of miR-9-5p and the percentage of CD4^+^IL-10^+^ T cells. Data represent the mean scores ± SEM of at least three independent experiments, ^*^p < 0.05, ^**^p < 0.01, Con group vs. AAA group; ^##^p < 0.01, ^#^p < 0.05, AAA+Ago group vs. AAA+Ago NC group.

The development of AAA is known to be significantly influenced by T-cell-mediated immunological responses (Téo et al., 2018). The expression of miR-9-5p in CD4^+^ T cells and the percentage of CD4^+^IL-10^+^ T cells were measured to evaluate the impact of miR-9-5p on the differentiation of CD4^+^IL-10^+^ T cells during the development of an AAA. The percentage of CD4^+^IL-10^+^ T cells and miR-9-5p expression were significantly lower in AAA lesion model animals than in control animals, as illustrated in Figures 3A–3C. Furthermore, compared with the agomir NC group, the miR-9-5p agomir intervention markedly increased the percentage of CD4^+^IL-10^+^ T cells and miR-9-5p expression. Additionally, a significant positive correlation between the percentage of CD4^+^IL-10^+^ T cells in AAA lesion model mice and the expression of miR-9-5p was detected by Pearson’s correlation analysis (r = 0.482, p = 0.031) (Figure 3D). These findings demonstrated that the expression of miR-9-5p in T cells during the development of an AAA may influence the differentiation of CD4^+^IL^−^10^+^ T cells.

### 3.4. miR-9-5p regulates the activity of the NF-κB–Nrf2 pathway in CD4^+^ T cells from AAA lesion model mice

**Figure 4:**
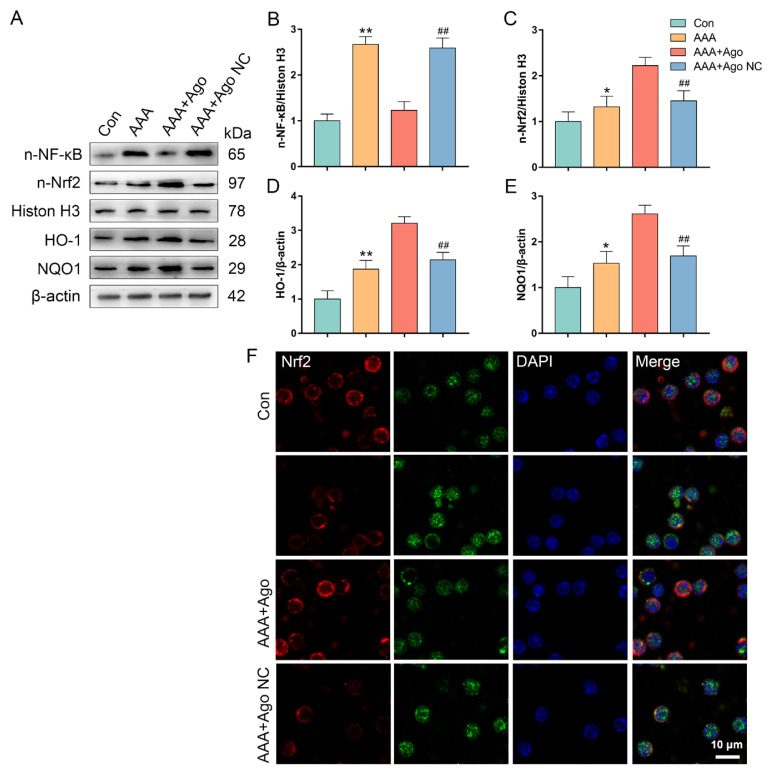
*miR-9-5p regulates the expressions of NF-κB-Nrf2 pathway of CD4^+^ T cells in AAA lesion mice*. (A) The protein expressions of the NF-κB-Nrf2 pathway and its downstream target genes were evaluated by western blot. (B) The protein level of nuclear NF-κB. (C) The protein level of nuclear Nrf2. (D) The protein level of HO-1. (E) The protein level of NQO1. (F) Nuclear translocation of Nrf2 and NF-κB were determined by immunofluorescent staining (bar = 10 μm). Data represent the mean scores ± SEM of at least three independent experiments, ^*^p < 0.05, ^**^p < 0.01, Con group vs. AAA group; ^##^p < 0.01, ^#^p < 0.05, AAA+Ago group vs. AAA+Ago NC group. NF-κB, nuclear factor kappaB; Nrf2, NF-E2-related factor 2; NQO1, NAD(P)H quinone oxidoreductase 1; HO-1, heme oxygenase-1.

According to our earlier research, the interaction between Nrf2 and NF-κB plays a role in controlling redox metabolism and CD4^+^ T-cell development (Zhang et al., 2021). Here, by identifying the expression of the NF-κB–Nrf2 pathway, we further investigated the possible mechanism by which miR-9-5p controls the metabolism of CD4^+^ T cells during AAA formation. When we compared AAA lesion model animals to control animals, we observed significantly greater expression of nuclear NF-κB and nuclear Nrf2 (Figures 4A–4C). However, nuclear NF-κB expression was dramatically downregulated, and nuclear Nrf2 expression was significantly increased by miR-9-5p agomir intervention (Figures 4A–4C). Furthermore, a similar pattern to that of n-Nrf2 before and after miR-9-5p intervention was observed in the expression of antioxidant genes downstream of Nrf2 (HO-1 and NQO1) (Figures 4D and 4E). Examining the nuclear localization of Nrf2 and NF-κB in the various groups further corroborated these findings (Figure 4F). Thus, the data showed that miR-9-5p regulates the expression of the NF-κB–Nrf2 pathway to alleviate the abnormal redox metabolism of CD4^+^ T cells in AAA lesion model mice.

### 3.5. miR-9-5p directly targets NF-κB

**Figure 5:**
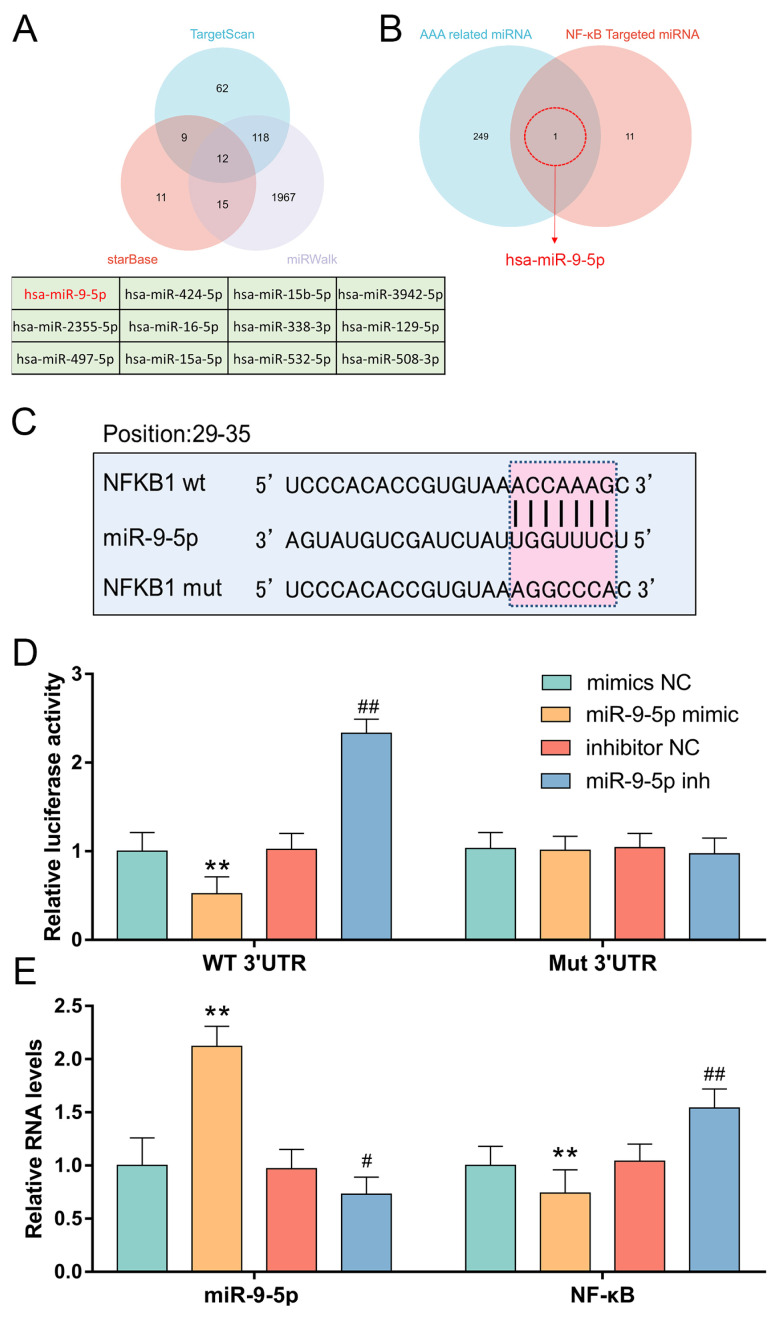
*miR-9-5p Directly Targets the NF-κB*. (A) Target miRNAs intersection analysis for NF-κB. (B) Analysis of intersections among AAA-related miRNAs and NF-κB target miRNAs. (C) Potential binding sites between miR-9-5p and NF-κB 3′UTR predicted by TargetScan. **(**D**)** Dual-luciferase assays showing repression of wild-type NF-κB -3′UTR following transfection of miR-9-5p mimics or negative control. **(**E**)** The levels of miR-9-5p and NF-κB mRNA in 293T cells after miR-9-5p overexpression and inhibition. Data represent the mean scores ± SEM of at least three independent experiments, ^*^p < 0.05, ^**^p < 0.01, Con group vs. AAA group; ^##^p < 0.01, ^#^p < 0.05, AAA+Ago group vs. AAA+Ago NC group. NF-κB, nuclear factor kappaB.

To verify that miR-9-5p is a suitable miRNA selected as a targeted inhibitor of NF-κB, a key molecule in the NF-κB–Nrf2 antioxidant pathway, we conducted relevant bioinformatics analysis. As shown in Figure 5A, 12 miRNAs capable of targeted regulation of NF-κB were predicted using the miRWalk, starBase and TargetScan databases. We subsequently carried out a cross-analysis of the 12 miRNAs targeted by NF-κB and the 250 miRNAs related to AAA in GeneCards^2^ and ultimately selected miR-9-5p (Figure 5B). TargetScan was used to identify putative binding sites between NF-κB 3′UTR sequences and miR-9-5p (Figure 5C). To determine whether miR-9-5p controls NF-κB expression by targeting its 3′UTR, a luciferase reporter system was constructed. Compared with the control, treatment with miR-9-5p mimics decreased luciferase activity, whereas treatment with miR-9-5p inhibitors significantly increased relative luciferase activity. Furthermore, when the seed sequence of the miR-9-5p binding sites was altered, the impact of miR-9-5p on luciferase activity was not observed (Figure 5D). Additionally, we altered the expression of miR-9-5p in 293T cells. Using qRT–PCR analysis, we showed that cells treated with the miR-9-5p mimics presented lower levels of NF-κB mRNA, whereas cells treated with NF-κB inhibitors presented opposite trends (Figure 5E). These findings suggest that miR-9-5p targets and adversely regulates NF-κB.

### 3.6. miR-9-5p promotes the differentiation of CD4^+^IL-10^+^ T cells by targeting the activity of the NF-κB-Nrf2 pathway in vitro

**Figure 6:**
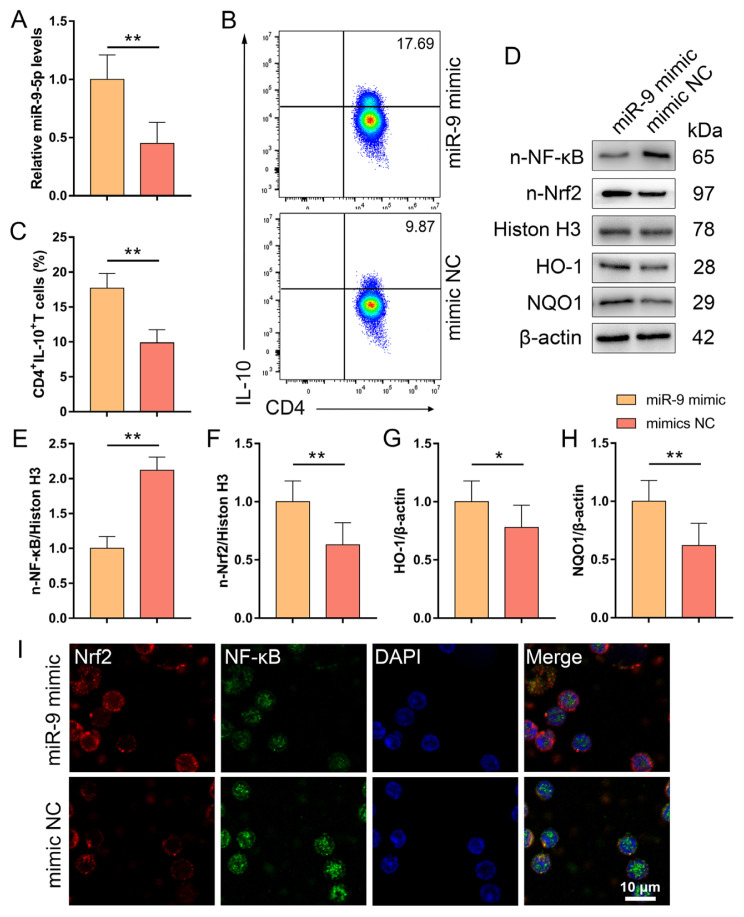
*miR-9-5p promotes the differentiation of CD4^+^IL-10^+^T cells by targeting the expressions of NF-κB-Nrf2 pathway in vitro*. (A) The expression of miR-9-5p. (B) Representative dot plots of CD4^+^IL-10^+^ T cells. (C) The percentage of CD4^+^IL-10^+^ T cells. (D-H) The protein expressions of the NF-κB-Nrf2 pathway and its downstream target genes. (I) Nuclear translocation of Nrf2 and NF-κB were determined by immunofluorescent staining (bar = 10 μm). All data are expressed as the mean ± SD, ^*^p < 0.05, ^**^p < 0.01, Con group vs. AAA group; ^##^p < 0.01, ^#^p < 0.05, AAA+Ago group vs. AAA+Ago NC group. NF-κB, nuclear factor kappaB; Nrf2, NF-E2-related factor 2.

We further verified whether the protective benefits observed in AAA lesion model animals treated with miR-9-5p were largely due to the NF-κB–Nrf2 pathway. The expression of n-Nrf2 and n-NF-κB was examined during the development of CD4^+^IL-10^+^ T cells from naive CD4^+^ T cells treated with the miR-9-5p mimic or mimic NC. Compared with the mimic NC group, the miR-9-5p mimic group presented significantly increased levels of miR-9-5p in CD4^+^ T cells, as shown in Figure 6A. Furthermore, the percentage of CD4^+^IL-10^+^ T cells in the miR-9-5p mimic group was significantly greater than that in the mimic NC group (Figures 6B and 6C). In CD4^+^ T cells, miR-9-5p mimic administration markedly reduced the expression of n-NF-κB and markedly increased the expression of n-Nrf2, HO-1, and NQO1 (Figures 6D–6I). These findings suggest that miR-9-5p controls the interaction between NF-κB and Nrf2 to promote the differentiation of CD4^+^IL-10^+^ T cells.

## 4. Discussion

Increased oxidative damage and inflammation are major causes of AAA pathological deterioration. CD4^+^ T-cell-based immune metabolic dysfunction may worsen AAA lesions. Our work revealed that miR-9-5p can modulate the NF-κB–Nrf2 axis in CD4^+^ T cells, improve the differentiation of CD4^+^IL-10^+^ T cells, and ameliorate AAA-related immunological dysfunction.

Local oxidative stress has a well-documented role in AAA development (McCormick et al., 2007; Papalambros et al., 2007). In the present study, we found that in AAA lesion model mice the serum oxidative stress levels were significantly greater and the antioxidant capacity was lower. Recent research has shown that human AAA tissue is distinguished by early infiltration of innate and adaptive inflammatory cells, which occurs prior to observable ECM degradation and an increase in aortic diameter (Potteaux and Tedgui, 2015; Zhang and Wang, 2015). This inflammatory environment significantly contributes to vascular oxidative stress, which may accelerate the recruitment of inflammatory cells in a reciprocal feedback loop (Zhang and Wang, 2015). Our study demonstrated that AAA lesion model mice presented considerably higher levels of proinflammatory cytokines (IL-6, TNF-α, and IFN-γ) and lower levels of antiinflammatory cytokines (IL-10). In addition, our findings suggest that miR-9-5p intervention can repair oxidative stress injury and proinflammatory cytokine bursts in AAA lesion model animals. Numerous studies have shown that aberrant lymphocyte metabolism and subgroup differentiation, represented by CD4^+^ T cells, are strongly associated with the initiation and progression of AAA (Koch et al., 1990; Raffort et al., 2017). Yin et al. (2010) reported a considerably lower level of FOXP3 expression in peripheral CD4^+^CD25^+^ T cells and a lower frequency of CD4^+^CD25^+^FOXP3^+^ T cells in patients with AAA. Similarly, we found that the fraction of CD4^+^IL-10^+^ T cells was much lower in AAA lesion model animals. Interestingly, miR-9-5p therapy increased the fraction of CD4^+^IL-10^+^ T cells in AAA lesion model animals, indicating a substantial positive connection. This finding is also consistent with the large increase in serum IL-10 levels following miR-9-5p treatment. The upregulation of IL-10, an important antiinflammatory cytokine, may be one of the reasons for the ability of miR-9-5p to reduce AAA-induced pathological damage.

Nrf2 and NF-κB are transcription factors that control cellular responses to oxidative stress and inflammation, respectively (Hayden and Ghosh, 2011; Wang et al., 2020; Xiong et al., 2020). The NF-κB pathway is considered an archetypal proinflammatory signaling route, partly due to its participation in the expression of proinflammatory genes such as cytokines, chemokines, and adhesion molecules (Hayden and Ghosh, 2011; Fan et al., 2013). Nrf2 is a transcription factor that controls several antioxidation and detoxification enzymes (Xiong et al., 2020). Pharmacological and genetic research has revealed that these two major pathways interact functionally. According to Wardyn et al. (2015), NF-κB activation inhibits Nrf2 by competing for the transcriptional coactivator CREB-binding protein (CBP)-p300 complex. A recent study revealed that Nrf2 is a novel regulator involved in maintaining the vascular smooth muscle cell contractile phenotype while influencing AAA formation through an miR-145-dependent regulatory mechanism (Xiao et al., 2024). In addition, many studies have reported that a variety of drugs can alleviate AAA by targeting and activating the Nrf2 antioxidant pathway to regulate the metabolism of smooth muscle cells and reduce the inflammatory response (Song et al., 2020; Wang et al., 2024; Zou et al., 2024). In this study, we observed significantly increased nuclear translocation of NF-κB and reduced nuclear translocation of Nrf2 in CD4^+^ T cells from AAA model mice. Concomitantly, the Nrf2 downstream antioxidant genes HO-1 and NQO1 were significantly downregulated, which correlated with the markedly elevated inflammatory response and oxidative stress levels in the AAA group. These findings suggest that abnormal expression of the NF-κB–Nrf2 axis represents a potential contributing factor to AAA pathogenesis. This dysregulation likely creates a vicious cycle: activated NF-κB promotes the production of proinflammatory cytokines (e.g., IL-6 and TNF-α), whereas suppressed Nrf2 impairs antioxidant defenses, collectively exacerbating vascular wall damage and remodeling.

miRNAs can lead to abnormal remodeling of the extracellular matrix, inhibition of the cell cycle, cellular senescence, or the exacerbation of inflammation. The role of miRNAs in the pathogenesis of abdominal aortic aneurysms and their potential as biomarkers have been widely explored recently (Borek et al., 2019). Yamasaki et al. (2022) reported that the inhibition of microRNA-33b can specifically ameliorate the formation of abdominal aortic aneurysms by suppressing the inflammatory pathway. A study by Zhang et al. (2020) showed that miR-146a regulates inflammation and development of abdominal aortic aneurysms in patients by targeting CARD10. Hu et al. (2022) reported that exosomal miR-17-5p derived from adipose-derived mesenchymal stem cells inhibits AAA by suppressing the TXNIP-NLRP3 inflammasome. Analysis of this study’s database and a dual-luciferase reporter test revealed that miR-9-5p can directly target NF-κB. In vitro tests confirmed that the miR-9-5p mimic effectively inhibited NF-κB expression in CD4^+^ T cells. Downregulating NF-κB expression led to increased expression of Nrf2 and downstream antioxidant genes (HO-1 and NQO1), as well as an increase in CD4^+^IL-10^+^ T cells. These findings support our previous reports that the interaction between Nrf2 and NF-κB signaling influences CD4^+^IL-10^+^ T-cell development (Zhang et al., 2021). These results suggest that miR-9-5p corrects the NF-κB–Nrf2 imbalance caused by AAA by targeting and inhibiting NF-κB, thereby increasing antioxidant capacity and reducing the release of inflammatory factors. Specifically, the miR-9-5p-mediated suppression of NF-κB nuclear translocation alleviates the competitive inhibition of Nrf2, leading to the upregulation of downstream antioxidant genes (e.g., HO-1 and NQO1) and decreased production of proinflammatory cytokines (e.g., IL-6 and TNF-α). This dual regulation of the redox–inflammatory axis highlights miR-9-5p as a potential therapeutic agent to restore vascular homeostasis during AAA by reestablishing the functional equilibrium between NF-κB and Nrf2.

## 5. Conclusions

Our findings revealed a possible new role for miR-9-5p in alleviating Ang II-induced AAA. miR-9-5p may regulate the crosstalk between Nrf2 and NF-κB in CD4^+^ T cells, leading to an increase in CD4^+^IL-10^+^ T cells. The findings of the present study suggest a novel strategy for developing a therapeutic method for patients with AAA-related immunological dysfunction.

## Supplementary material

**Table:** Sequences used for RT-qPCR

Name	Primer sequences (forward/reverse primer)
**miR-9-5p**	CGAGCTCTGTGTGTGTGTGTGTGTG TTCCGCGGCCGCTATGGCCGAGGAA
**NF-κB**	CTCCGAGACTTTCGAGGAAATAC GCCATTGTAGTTGGTAGCCTTCA
**U6**	CTCGCTTCGGCAGCACA AACGCTTCACGAATTTGCGT
**β-actin**	CACCACACCTTCTACAATGAG TACGACCAGAGGCATACAG

## Data Availability

The datasets used and/or analyzed during the current study are available from the corresponding author upon reasonable request.

## Footnotes

1 TargetScan [online]. Website: http://www.targetscan.org [accessed 09 July 2025].

2 GeneCards [online]. Website: https://www.genecards.org/ [accessed 09 July 2025].
