# Corrigendum to “miR-9-5p alleviates the development of abdominal aortic aneurysm by regulating the differentiation of CD4^+^IL-10^+^T cells via targeting the crosstalk between Nrf2 and NF-κB signaling pathways” [Turkish Journal of Biology 49 (4) 2025 380–391]

**DOI:** 10.55730/1300-0152.2784

**Published:** 2025-12-13

**Authors:** Hongfu LIU, Jinyi ZHANG, Lubin LI, Benxiang YU, Chunlei ZHANG, Wenqiang NIU, Yawen CHENG, Hengyang DONG, Yukun ZHANG, Xinlin LUO, Yanlian XIONG, Yueming WANG

**Affiliations:** 1 Department of Anatomy, School of Basic Medicine, Binzhou Medical University, Yantai, Shangdong Province, China; 2 Xu Rongxiang Regenerative Medicine Research Center, Binzhou Medical University, Yantai, P.R. China; 3 Department of Vascular Surgery, Yantai Yuhuangding Hospital, Yantai, Shangdong Province, China

This corrigendum is to address an issue regarding the manuscript’s previous publication. The authors noticed that the representative flow cytometry image for the AAA group in Figure 3B, as well as the representative Histone H3 and β-actin Western blot bands in Figure 4A, were incorrectly used in the original published version of this paper and indicated that the relevant corrections do not affect the results or the conclusions of the study.

To rectify this oversight and ensure the accuracy of the published work, the correct figures are included for your reference.

DOI of original article: https://doi.org/10.55730/1300-0152.2754

Correspondence: xyl8807@163.com, wym15@bzmc.edu.cn

https://doi.org/10.55730/1300-0152.2784

**Figure 3:**
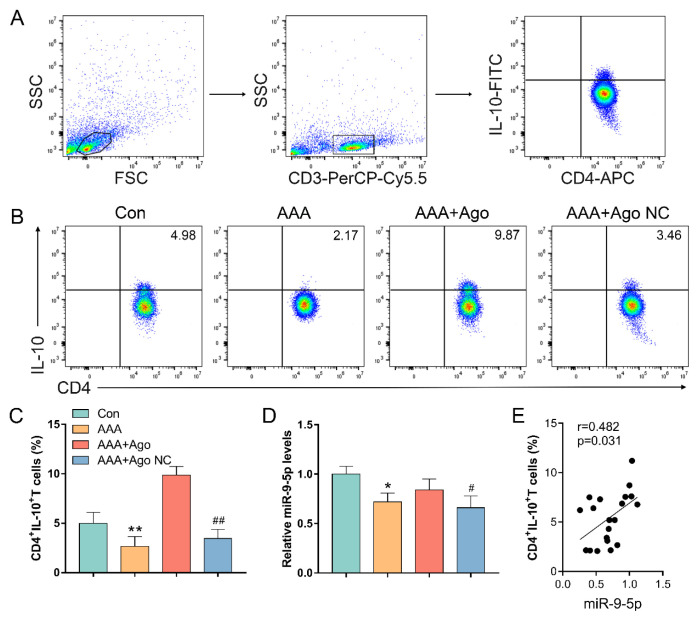
*miR-9-5p regulates the differentiation of CD4* *^+^* *IL-10* *^+^* *T cells in AAA lesion mice* (A) Gating strategy: forward scatter (FSC) and side scatter (SSC) gating were used to discriminate viable cells from cell debris. Within the lymphocyte gate, CD3 was used as a T cell maker. (B) Representative dot plots of CD4^+^IL-10^+^ T cells. (C) The percentage of CD4^+^IL-10^+^ T cells. (D) The expression of miR-9-5p. (E) Pearson’s correlation analysis between the levels of miR-9-5p and the percentage of CD4^+^IL-10^+^ T cells. Data represent the mean scores ± SEM of at least three independent experiments, ^*^p < 0.05, ^**^p < 0.01, Con group vs. AAA group; ^##^p < 0.01, ^#^p < 0.05, AAA+Ago group vs. AAA+Ago NC group.

**Figure 4:**
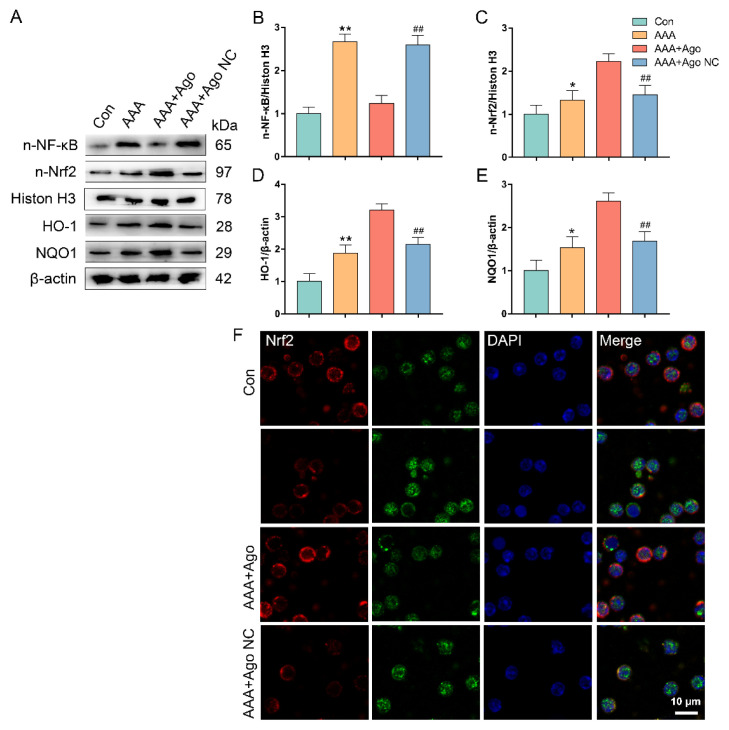
*miR-9-5p regulates the expressions of NF-κB-Nrf2 pathway of CD4* *^+^* *T cells in AAA lesion mice* (A) The protein expressions of the NF-κB-Nrf2 pathway and its downstream target genes were evaluated by western blot. (B) The protein level of nuclear NF-κB. (C) The protein level of nuclear Nrf2. (D) The protein level of HO-1. (E) The protein level of NQO1. (F) Nuclear translocation of Nrf2 and NF-κB were determined by immunofluorescent staining (bar = 10 μm). Data represent the mean scores ± SEM of at least three independent experiments, ^*^p < 0.05, ^**^p < 0.01, Con group vs. AAA group; ^##^p < 0.01, ^#^p < 0.05, AAA+Ago group vs. AAA+Ago NC group. NF-κB, nuclear factor kappaB; Nrf2, NF-E2-related factor 2; NQO1, NAD(P)H quinone oxidoreductase 1; HO-1, heme oxygenase-1.

